# Molecular Subtype May Be More Associated With Prognosis and Chemotherapy Benefit Than Tumor Size in T1N0 Breast Cancer Patients: An Analysis of 2,168 Patients for Possible De-Escalation Treatment

**DOI:** 10.3389/fonc.2021.636266

**Published:** 2021-02-19

**Authors:** Siji Zhu, Yafen Li, Weiguo Chen, Xiaochun Fei, Kunwei Shen, Xiaosong Chen

**Affiliations:** ^1^ Department of General Surgery, Comprehensive Breast Health Center, Ruijin Hospital, Shanghai Jiaotong University School of Medicine, Shanghai, China; ^2^ Department of Pathology, Ruijin Hospital, Shanghai Jiaotong University School of Medicine, Shanghai, China

**Keywords:** breast cancer, molecular subtype, prognosis, chemotherapy benefit, de-escalating therapy

## Abstract

**Purpose:**

Breast cancer (BC) patients with T1N0 tumors have relatively favorable clinical outcomes. However, it remains unclear whether molecular subtypes can aide in prognostic prediction for such small, nodal-negative BC cases and guide decision-making about escalating or de-escalating treatments.

**Patients and Methods:**

T1N0 BC patients diagnosed between 2009 and 2017 were included and classified into three subgroups according to receptor status: 1) hormonal receptor (HR)+/human epidermal growth factor receptor-2 (HER2)−; 2) HER2+; and 3) triple negative (TN) (HR−/HER2−). Patients’ characteristics and relapse events were reviewed. Kaplan–Meier analysis and Cox regression were used to assess the iDFS and BCSS. The effects of risk factors and adjuvant treatment benefits were evaluated by calculating hazard ratios (HRs) for invasive disease-free survival (iDFS) and breast cancer-specific survival (BCSS) with Cox proportional hazards models.

**Results:**

In total, 2,168 patients (1,435 HR+/HER2−, 427 HER2+, 306 TN) were enrolled. The 5-year iDFS rates were 93.6, 92.7, and 90.6% for HR+/HER2−, HER2+, and TN patients, respectively (P = 0.039). Multivariate analysis demonstrated that molecular subtype (P = 0.043), but not tumor size (P = 0.805), was independently associated with iDFS in T1N0 BC. TN patients [HRs = 1.77, 95% confidence interval (CI) = 1.11–2.84, P = 0.018] had a higher recurrence risk than HR+/HER2− patients. Adjuvant chemotherapy benefit was not demonstrated in all T1N0 patients but interacted with molecular subtype status. TN (adjusted HRs = 2.31, 95% CI = 0.68–7.54) and HER2+ (adjusted HRs = 2.26, 95% CI = 0.95–5.63) patients receiving chemotherapy had superior iDFS rates. Regarding BCSS, molecular subtype tended to be related to outcome (P = 0.053) and associated with chemotherapy benefit (P = 0.005).

**Conclusion:**

Molecular subtype was more associated with disease outcome and chemotherapy benefit than tumor size in T1N0 BC patients, indicating that it may guide possible clinical de-escalating therapy in T1N0 BC.

## Introduction

With the rise of breast cancer (BC) awareness and mammographic screening over the past decade, T1N0 BC has been diagnosed with increasing frequency (1). Generally, these early-stage BC patients are considered to have an excellent long-term outcome after surgical operation (2, 3). Thus, most previous studies focused on larger nodal-negative and axillary nodal-positive BC patients, for whom their recurrence risk requires aggressive management. In the current staging system, of which clinicopathological prognostic factors such as tumor size and regional node status are the basis, T1N0 BC tumors are all placed into a generally low recurrence risk group, and cannot distinguish their intrinsic prognostic difference (4). However, even small BC tumors can exhibit aggressive behavior. Previous studies have shown that BC relapse and deaths occur in these small BC patients, and nearly 1/4 of all recurrences occur beyond 10 years (5–7). Furthermore, the low representation of T1N0 tumor patients in those studies leads to a lack of high-level evidence to guide clinicians in the treatment of these patients, especially for the administration and benefit of chemotherapy for T1N0 BCs.

Over the last few decades, our understanding of BC tumors has improved dramatically. The emergence of tumor biology has enabled us to understand why patients with similar stages have significantly different outcomes and different responses to adjuvant systemic agents. Molecular subtypes, defined by estrogen receptor (ER), progesterone receptor (PgR), and human epidermal growth factor receptor-2 (HER2) status, can classify BCs into three different subgroups [luminal-like, triple negative (TN), and HER2 positive] and are important for predicting prognosis and treatment benefits for breast cancer (8, 9). However, cause T1N0 tumor were usually excluded from those previous clinical studies, it is still uncertain whether molecular subtypes can aid in prognostic prediction for such small, nodal-negative BC cases and guide decision-making about clinical escalating or de-escalating treatments.

Based on the above issues, we conducted this study to evaluate the associations of tumor biology and prognosis as well as chemotherapy benefit in T1N0 BC patients, thus guiding further clinical individualized therapy.

## Patients and Methods

### Study Population

Female patients who underwent surgery for invasive BC at Ruijin Hospital were retrospectively included. All BC patients with T1N0 tumors between Jan. 2009 and Dec. 2017 were identified through the Shanghai Jiaotong University Breast Cancer Database (SJTU-BCDB). The collected data included patients’ characteristics [e.g., age, menopausal status, tumor size, pathological type, histologic grade, hormonal receptor(HR), HER2 status] and details of treatment (e.g., breast surgery, radiotherapy, chemotherapy, endocrine therapy, and HER2-targeted therapy). The definitions of T1a, T1b, and T1c were based on the seventh edition American Joint Committee on Cancer(AJCC) TNM staging system (4). If patients had primary metastatic disease, received neoadjuvant systemic treatment, or already had a personal history of BC, they were excluded from this study.

Hormonal receptor (ER/PgR) was defined as positive if the tumor had at least 1% nuclear staining by immunohistochemistry (IHC) techniques (10). HER2 positivity was determined as IHC HER2 3+ or positive on fluorescence *in situ* hybridization (FISH) (11). According to the HR and HER2 status, all patients were divided into three subtypes: 1) HR+/HER2− (ER+ or PgR+, HER2−); 2) HER2+(HR+/−, and HER2+); and 3) triple negative (TN) (HR−, and HER2−).

### Follow-Up

For all patients, outpatient visits or calls were performed every 3 to 6 months until death. Invasive disease-free survival (iDFS) was defined as the length of time from primary surgery to the first occurrence of the following events: any invasive disease of locoregional recurrence, contralateral invasive BC, distant recurrence, secondary non-breast malignant tumors, and any cause of death. Breast cancer-specific survival (BCSS) was defined as the length of time from primary surgery to BC-related death.

### Statistical Analysis

Pearson’s chi-square test was used to compare the clinicopathological features and treatment choices among different groups. Kaplan–Meier analysis and multivariable Cox regression were used to assess the iDFS and BCSS. The impact of different prognostic factors on iDFS and BCSS, as well as interactions between chemotherapy benefit and those prognostic factors, were examined by Cox proportional hazards regression. Two-sided P values<0.05 were considered statistically significant. Statistical analysis procedures were conducted with IBM SPSS version 20 (SPSS Inc., Chicago, IL, USA).

## Results

### Basic Characteristics and Clinicopathological Factors of the Subtypes

**Figure 1:**
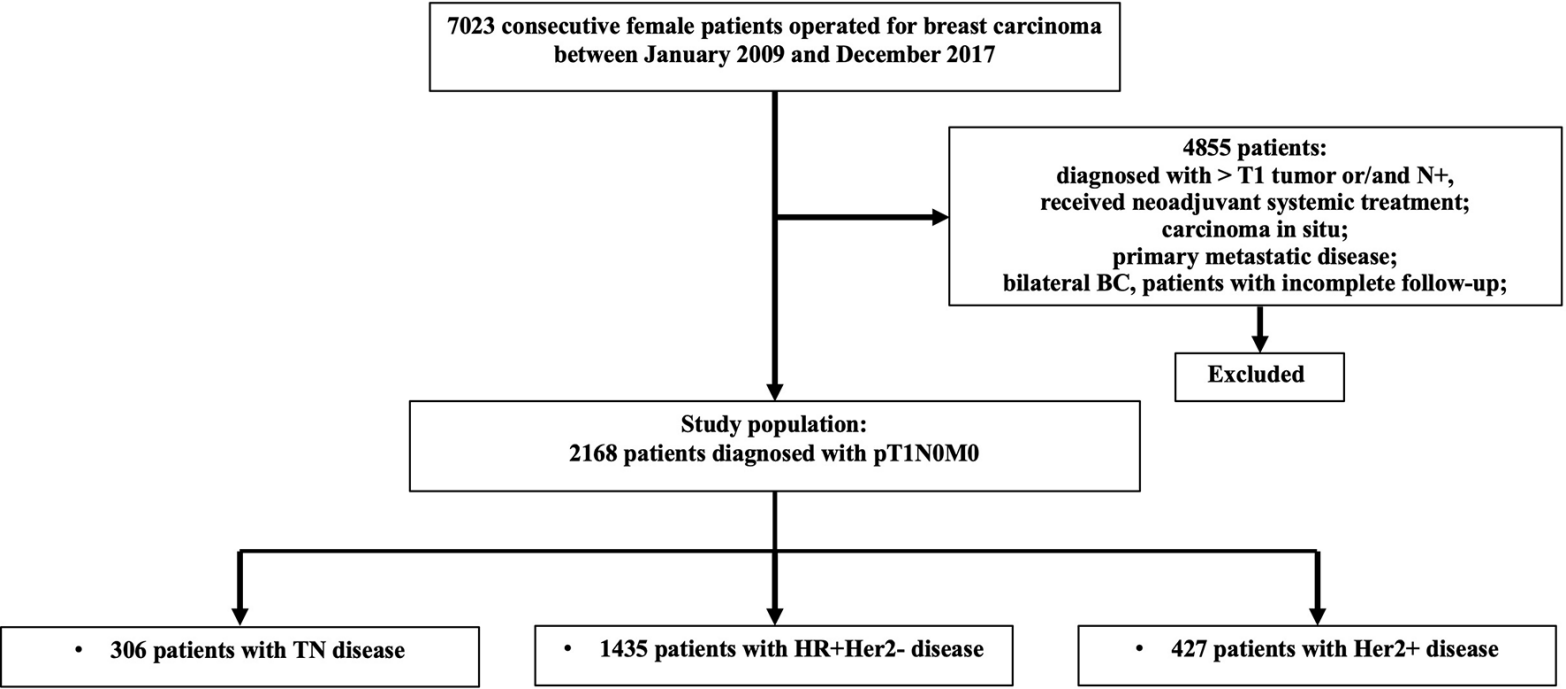
Identification of the study population.

Among 7,023 patients with breast cancer who received surgery between 2009 and 2017, 2,168 pT1N0 patients were included (**Figure 1**). **Table 1** shows the patients’ characteristics. The median age was 56 (26–91) years, and a total of 1,382 (63.7%) patients were postmenopausal. Overall, 66.2% of patients were classified as HR+/HER2−, 19.7% as HER2+, and 14.1% as triple-negative breast cancer (TNBC). Regarding tumor size, 344 (15.9%), 457 (21.1%), and 1,367 (63.1%) patients were T1a, T1b, and T1c, respectively.

**Table 1:** Patients’ characteristics.

Characteristics	ALLN (%) = 2,168	HR+/HER2−N (%) = 1,435	HER2+N (%) = 427	TNN (%) = 306	P
Age					<0.001
<50 50–65 65+	701 (32.3%) 986 (45.5%) 481 (22.2%)	444 (30.9%) 618 (43.1%) 373 (26.0%)	160 (37.5%) 224 (52.5%) 43 (10.1%)	97 (31.7%) 144 (47.1%) 65 (21.2%)	
Menopausal status					0.005
Premenopausal Postmenopausal	786 (36.3%) 1,382 (63.7%)	509 (35.5%) 926 (64.5%)	181 (42.4%) 246 (57.6%)	96 (35.2%) 210 (68.6%)	
Breast surgery type					<0.001
Lumpectomy Mastectomy	847 (39.1%) 1,321 (60.9%)	595 (41.5%) 840 (58.5%)	123 (28.8%) 304 (71.2%)	129 (42.2%) 177 (57.8%)	
Pathological type					<0.001
IDC ILC Others	1,852 (85.4%) 75 (3.5%) 241 (11.1%)	1,187 (82.7%) 59 (4.1%) 189 (13.2%)	402 (94.1%) 7 (1.6%) 18 (4.2%)	263 (85.9%) 9 (2.9%) 34 (11.1%)	
Tumor size					<0.001
T1a T1b T1c	344 (15.9%) 457 (21.1%) 1,367 (63.1%)	163 (11.4%) 354 (24.7%) 918 (64.0%)	122 (28.6%) 59 (13.8%) 246 (57.6%)	59 (19.3%) 44 (14.4%) 203 (66.3%)	
Histological grade					<0.001
I II III NA	170 (7.8%) 1,012 (46.7%) 565 (26.1%) 421 (19.4%)	161 (11.2%) 773 (53.9%) 223 (15.5%) 278 (19.4%)	6 (1.4%) 152 (35.6%) 187 (43.8%) 82 (19.2%)	3 (1.0%) 87 (28.4%) 155 (50.7%) 61 (19.9%)	
Ki67					<0.001
<14% ≥14%	1,016 (46.9%) 1,152 (53.1%)	852 (59.4%) 583 (40.6%)	88 (20.6%) 339 (79.4%)	76 (24.8%) 230 (75.2%)	

IDC, invasive ductal carcinoma; ILC, invasive lobular carcinoma; HR, hormonal receptor; HER2, human epidermal growth factor receptor-2; TN, triple negative; NA, not available.

HER2+ BC accounted for 28.6% of T1a tumors, which was higher than the proportions of TNBC (19.3%) and HR+/HER2− (11.4%) (P<0.001). More elderly patients (65+) were found in the HR+/HER2− group (26.0%) than in the TN (21.2%) and HER2+ (10.1%) groups (P<0.001). Regarding tumor grade, 65.1% of the HR+/HER2− group were grade II or III, which was much lower than the proportions in the TN group (79.1%) or the HER2+ group (79.4%) (P<0.001). A similar result was found for the Ki67 level: 59.4% of HR+/HER2− patients had low Ki67 levels, and the proportions were only 20.6% for HER2+ patients and 24.8% for TN patients (P<0.001).

### Adjuvant Treatment and Associated Factors

In total, 1,080 (49.8%) patients were treated with adjuvant chemotherapy (**Table 2**). In univariate analysis, age, menopausal status, tumor grade, pathological type, tumor size, ER, PgR, HER2, Ki67, and molecular subtype were all found to be significantly associated with chemotherapy administration (P<0.001) (**Supplementary Table 1**). In multivariate analysis, we found that age, grade, tumor size, Ki67 level, and molecular subtype (P <0.001) were independent factors for chemotherapy administration. The median age (52 years) of patients receiving chemotherapy was significantly younger than those not receiving it (60 years) (P< 0.001), and fewer elderly patients (65+) underwent chemotherapy [OR = 0.10, 95% confidence interval (CI) = 0.07–0.15, P< 0.001]. Regarding patients’ clinicopathological features, more patients with large lesions (compared with T1a, T1b: OR = 9.52, 95% CI = 5.99–15.15; T1c: OR = 16.13, 95% CI = 10.53–25.00, P<0.001), high tumor grades (compared with grade I, grade II: OR = 1.97, 95% CI = 1.23–3.14, P = 0.005; grade III: OR = 3.61, 95% CI = 2.13–6.14, P<0.001), and high Ki67 levels (compared with Ki67 <14%, Ki67 ≥14%: OR = 6.37, 95% CI = 5.29–7.69, P<0.001) were given adjuvant chemotherapy. In terms of molecular subtype, HER2+ (80.1%) and TN (75.5%) BC patients were more likely (*vs*. HR+/HER2−, 35.3%) to receive adjuvant chemotherapy (HER2+: OR = 12.67, 95% CI = 8.77–18.52; TN, OR = 6.67, 95% CI = 4.67–9.52; P<0.001). Additionally, pathological type (P = 0.165) and menopausal status (P = 0.859) were not independent factors for chemotherapy administration in multivariate analysis. The detailed regimens information for chemotherapy are shown in **Supplementary Table 2**.

**Table 2:** Multivariate analyses of chemotherapy administration according to tumor characteristics.

Characteristics	Chemotherapy		Multivariate OR (95％CI)	P
	YES (N = 1,080)	NO (N = 1,088)	
Age (median age)	52	60		<0.001
<50 50–65 65+	422 (60.2%) 554 (56.2%) 104 (21.6%)	279 (39.8%) 432 (43.8%) 377 (78.4%)	1 0.86 (0.67–1.10) 0.10 (0.07–0.15)	0.233 <0.001
Menopausal status			0.859
Premenopausal Postmenopausal	465 (59.2%) 615 (44.5%)	321 (40.8%) 767 (55.5%)	1 0.97 (0.68–1.38)	
Histological grade			<0.001
I II III NA	32 (18.8%) 480 (47.4%) 459 (81.2%) 109 (25.9%)	138 (81.2%) 532 (52.6%) 106 (18.8%) 312 (74.1%)	1 1.97 (1.23–3.14) 3.61 (2.13–6.14) 0.73 (0.43–1.24)	0.005 <0.001 0.242
Pathological type			0.165
IDC ILC Others	994 (53.7%) 25 (33.3%) 61 (25.3%)	858 (46.3%) 50 (66.7%) 180 (74.7%)	1 1.59 (0.76–3.33) 0.80 (0.46–1.40)	0.217 0.440
Tumor size				<0.001
T1a T1b T1c	85 (24.7%) 188 (41.1%) 807 (59.0%)	259 (75.3%) 269 (58.9%) 560 (41.0%)	1 9.52 (5.99–15.15) 16.13 (10.53–25.00)	<0.001 <0.001
ER status			/^a^	
Positive Negative	666 (41.0%) 414 (76.1%)	958 (59.0%) 130 (23.9%)		
PgR status			/^a^	
Positive Negative	490 (36.4%) 590 (72.0%)	858 (63.6%) 230 (28.0%)		
HER2 status			/^a^	
Positive Negative	342 (80.1%) 738 (42.4%)	85 (19.9%) 1,003 (57.6%)		
Ki67 level				<0.001
<14% ≥14%	273 (26.9%) 807 (70.1%)	743 (73.1%) 345 (29.9%)	1 6.37 (5.29–7.69)	<0.001
Molecular subtype				<0.001
HR+/HER2- HER2+ TN	507 (35.3%) 342 (80.1%) 231 (75.5%)	928 (64.7%) 85 (19.9%) 75 (24.5%)	1 12.67 (8.77–18.52) 6.67 (4.67–9.52)	<0.001 <0.001

IDC, invasive ductal carcinoma; ILC, invasive lobular carcinoma; HR, hormonal receptor; HER2, human epidermal growth factor receptor-2; TN, triple negative; ER, estrogen receptor; PgR, progesterone receptor.

a Cause ER, PgR, and HER2 are components of molecular subtype, we included molecular subtype as an integral factor into multivariate analysis.

In total, 777 (35.8%) patients received radiotherapy after breast-conserving therapy, and 1,549 (94.5%) patients with HR+ disease received adjuvant endocrine therapy. For HER2+ BC patients, 275 (64.4%) received adjuvant trastuzumab treatment. Compared with T1a HER2+ patients (37.7%), T1b (69.5%, P<0.001), and T1c (76.4%, P<0.001) patients were more likely to be given trastuzumab.

### Disease Outcomes

**Figure 2:**
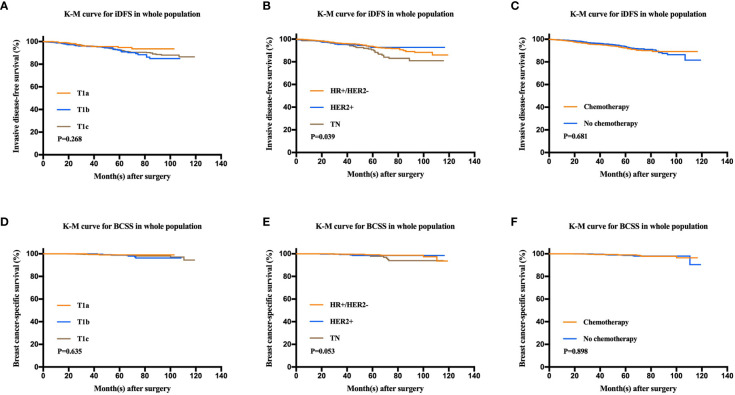
Kaplan-Meier analysis for invasive disease-free survival (iDFS) and breast cancer-specific survival (BCSS) according to tumor size, molecular subtype, and chemotherapy administration. **(A)** iDFS of T1a, T1b, and T1c tumors (P = 0.268). **(B)** iDFS of the HR+/HER2−, HER2+, and TN groups (P = 0.039). **(C)** iDFS of patients who received chemotherapy and those who did not (P = 0.681). **(D)** BCSS of T1a, T1b, and T1c tumors (P = 0.635). **(E)** BCSS of the HR+/HER2−, HER2+, and TN groups (P = 0.053). **(F)** BCSS of patients who received chemotherapy and those who did not (P = 0.898).

After a median follow-up of 47.9 months, 136 patients had iDFS events. The estimated 5-year iDFS rate was 93.0% in the whole population. Univariate analysis did not find significant differences of iDFS rates among patients with different ages, menopausal statuses, pathological types, tumor grades, tumor sizes, HER2 statuses, or Ki67 levels (P>0.05) (**Table 3**). The estimated 5-year iDFS rates were 94.8, 92.6, and 92.7% for the T1a, T1b, and T1c groups, respectively (P = 0.268) (**Figure 2A**). However, univariate analysis showed that ER, PgR, and molecular subtype were significantly correlated with iDFS in T1N0 patients (**Table 3**). The estimated 5-year iDFS rates were 93.6, 92.7, and 90.6% for HR+/HER2−, HER2+, and TN tumors, respectively, which showed a significantly better prognosis in the HR+/HER2− and HER2+ groups (P = 0.039) (**Figure 2B**). Multivariate analysis, including age, pathological type, tumor grade, tumor size, Ki67 level, and molecular subtype, showed that molecular subtype was the only prognostic factor for iDFS (P = 0.043). TN group patients had a significantly worse iDFS than HR+/HER2− group patients (HRs = 1.77; 95% CI = 1.11–2.84, P = 0.018), while no significant difference was found between the HER2+ and HR+/HER2− groups (HRs = 0.87; 95% CI = 0.62–1.77).

**Table 3:** Univariate analyses of invasive disease-free survival (iDFS) and breast cancer-specific survival (BCSS) according to tumor characteristics.

Factor	iDFS*P*	BCSS*P*
Age	0.464	0.180
≤50 *vs*. 50–65 65+ *vs*. ≤50	0.919 0.319	0.577 0.096
Menopausal status		
Pre- *vs*. post-menopausal	0.161	0.232
Pathological type	0.856	0.525
IDC *vs*. ILC IDC *vs*. others	0.674 0.696	0.978 0.257
Grade	0.761	0.192
I *vs*. II I *vs*. III	0.427 0.527	0.627 0.379
Tumor size	0.273	0.641
T1a *vs*. T1b T1a *vs*. T1c	0.108 0.239	0.367 0.567
ER status		
Positive *vs*. negative	0.011	0.010
PgR status		
Positive *vs*. negative	0.003	0.021
HER2 status		
Positive *vs*. negative	0.371	0.806
Ki67 level		
<14% *vs*. ≥14%	0.059	0.163
Molecular subtype	0.039	0.053
HR+/HER2− *vs*. HER2 HR+/HER2− *vs*. TN	0.686 0.020	0.773 0.020

Uv, univariate; iDFS, invasive disease-free survival; IDC invasive ductal carcinoma; ILC, invasive lobular carcinoma; HR, hormonal receptor; HER2, human epidermal growth factor receptor-2; TN, triple negative; BCSS, breast cancer-specific survival; ER, estrogen receptor; PgR, progesterone receptor.

There were 24 patients with BCSS events, with an estimated 5-year BCSS rate of 98.8% (95% CI = 98.22–99.38%). There was no significant difference of BCSS rates among patients with different tumor sizes (P = 0.635) (**Figure 2D**). In univariate analysis, age, menopausal status, pathological type, tumor grade, tumor size, HER2 status, and Ki67 level were not significantly associated with BCSS (P>0.05) (**Table 3**). Molecular subtype had a trend of significant BCSS difference (P = 0.053), with estimated 5-year BCSS rates of 99.1, 98.5, and 97.8% for the HR+/HER2−, HER2+, and TN groups, respectively (**Figure 2E**). Furthermore, the annual risk curve of iDFS and BCSS are shown in **Supplementary Figure 1**, which show a low annual recurrence risk of 1–2% for HR+/HER2− and HER2 patients, but small TNBC tumors had a recurrence peak at almost 5 years after surgery. 

### Factors Associated With Chemotherapy Benefit

Among the whole population, 1,080 (49.8%) patients received adjuvant chemotherapy. There were no differences in the iDFS or BCSS rates between patients receiving and not receiving chemotherapy. The 5-year iDFS rates were 93.6% for patients without chemotherapy and 92.4% for patients who received chemotherapy (P = 0.681). Similarly, the 5-year BCSS rates were 98.5% for patients without chemotherapy and 99% for patients receiving chemotherapy (P = 0.898) (**Figures 2C, F**).

**Figure 3:**
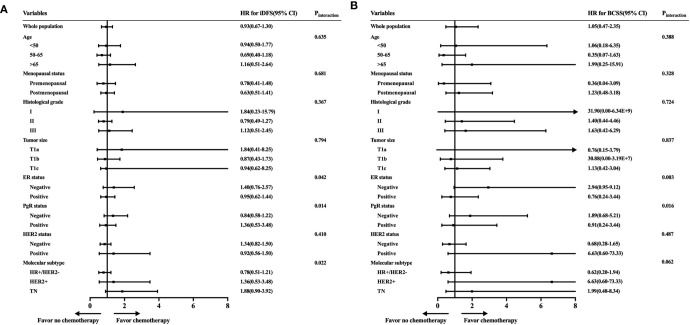
Exploratory analyses of invasive disease-free survival (iDFS) and breast cancer-specific survival (BCSS) rates according to patient characteristics and tumor subtype. **(A)** Forest plot of the hazard ratios of the iDFS rates of patients receiving chemotherapy compared with patients not receiving chemotherapy. **(B)** Forest plot of the hazard ratios of the BCSS rates of patients receiving chemotherapy compared with patients not receiving chemotherapy.

To further identify the patient population that can be managed with de-escalating treatments, the estimated HRs of the iDFS and BCSS rates for 2,168 women receiving or not receiving adjuvant chemotherapy were evaluated and are shown in **Figure 3**. When we investigated the iDFS benefit of chemotherapy according to clinicopathological features, the interaction between molecular subtype and chemotherapy was statistically significant (P_interaction_ = 0.022). Subgroup analysis showed that TN patients with chemotherapy had a lower recurrence risk than patients not receiving chemotherapy, with an iDFS HRs of 1.88, but the 95% CI did not rule out a meaningful difference (95% CI = 0.90–3.92) (**Figure 3A**). Similarly, a trend of iDFS benefit was observed for HER2+ patients who received chemotherapy, but this association was not statistically significant (HRs = 1.36, 95% CI = 0.53–3.48). Similar results were found based on ER status (P_interaction_ = 0.042) and PgR status (P_interaction_ = 0.014). We further analyzed BCSS, where the CIs were very wide due to the small number of deaths in each of the subpopulations (**Figure 3B**). A marginal interaction was seen according to molecular subtype (P_interaction_ = 0.062), and ER/PgR status was statistically significant (P_interaction_ <0.05).

**Figure 4:**
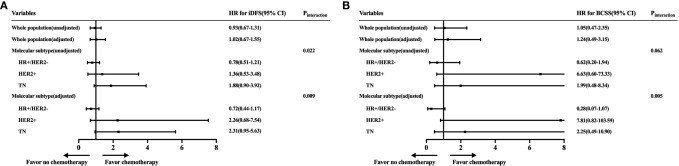
Forest plot of unadjusted and adjusted hazard ratios of invasive disease-free survival (iDFS) and breast cancer-specific survival (BCSS) rates according to molecular subtype. The unadjusted estimates were from the Cox models with only the exposures of interest. The adjusted models were estimated by incorporating factors that would influence a clinician’s decision to offer adjuvant chemotherapy: molecular subtype, age, tumor grade, and tumor size.

Furthermore, the adjusted HRs of iDFS and BCSS rates with incorporating factors that would influence adjuvant chemotherapy administration were shown (**Figure 4**). Chemotherapy did not have survival benefit among whole population in the adjusted Cox models. The adjusted HRs were 1.02 (95% CI = 0.67–1.55) for iDFS and 1.24 (95% CI = 0.49–3.15) for BCSS between patients receiving and not receiving chemotherapy. However, the interactions between molecular subtype and chemotherapy were still statistically significant in the adjusted Cox models (iDFS: P_interaction_ = 0.009; BCSS: P_interaction_ = 0.005).

## Discussion

With the increasing incidence of small BC due to early detection, the question of which treatment is appropriate for these patients emerges. Adjuvant chemotherapy decisions are usually based on traditional clinicopathological factors, such as tumor size and nodal involvement, but its value in T1N0 tumors is challenging due to the good prognosis and the limited evidence from clinical trials. In the current study, we included 2,168 women with T1N0 tumors to evaluate which factors were more associated with disease outcomes and chemotherapy benefit. In this large cohort study, compared with tumor size, molecular subtype was more related to disease outcomes as well as chemotherapy benefit in T1N0 patients, which may help guide further clinical de-escalating therapy.

Our study found that among small BC patients, nearly two-thirds of T1N0 patients had HR+/HER2− tumors, approximately 60% of patients were postmenopausal, and 8% of patients had low-grade tumors, similar to the findings in other studies including small BC (12, 13). Among the whole T1N0 population, 2,168 patients had a good prognosis, with 5-year iDFS rates of 93.0% and BCSS of 98.8%. This result corresponds approximately to those in previous literatures. One study including patients from various NSABP trials reported a good prognosis for T1a, bN0 patients, with an 8-year overall survival (OS) of 92%, and in which BC attributed to half of the deaths (14). Another retrospective study from France also showed a 10-year OS of 90.7% for those T1N0 patients (6).

Our study did not find a significant difference in the iDFS and BCSS rates among women with T1a, T1b, or T1c tumors, indicating that tumor size was not a main determinant prognostic factor for T1 patients. These results are in accordance with several other literatures, and the relatively poor outcomes of T1a patients could be explained by low rates of adjuvant treatment administration and the presence of non-invasive components (15, 16). Based on our analyses, molecular subtype was a significant prognostic factor for T1N0 patients, women with TN tumors had the lowest survival rate, and those HR+/HER2− patients had the best prognosis. This finding is in line with those of several other studies and suggest that it is important to develop new innovative therapies even for patients with small TNBC tumors (17, 18). Regarding the HER2+ group, several previous literatures reported that HER2 overexpression was an important risk factor for early relapse in those small BCs (19, 20). However, our series revealed that HER2 positivity was not associated with worse prognosis. The first reason may be that most HER2+ BCs in our cohort received chemotherapy with or without trastuzumab, which might conceal the adverse effects of HER2 positivity. Furthermore, our data suggest that ER/PgR status might be a more important prognostic factor than HER2 status for T1N0 BC. For example, in multivariate analysis, if we assessed ER/PgR and HER2 status independently instead of molecular subtype, ER negativity was significantly associated with early relapse (iDFS: HRs = 1.748; 95% CI = 1.221–2.502; P = 0.002), but HER2 overexpression was not (data not shown in the *Results*). Regarding recurrence risk curve, our result showed a generally low annual recurrence risk for small BC, especially for HR+/HER2− or HER2+ group. The difference of recurrence risk curve for small BC compared with whole BC population, especially small TNBC with a mid-late recurrence peak, might explained by insufficient adjuvant chemotherapy to those small TNBC. 

When making decisions about adjuvant chemotherapy, medical oncologists should weigh the absolute benefit of treatment against the potential chemotherapy-related risks (e.g., infection, cardiomyopathy, neuropathy, secondary leukemias, and chemotherapy-related death) (21, 22). The absolute benefit of treatment was determined by the baseline risk of recurrence and the effect of treatment on the baseline prognosis, with tumor size, and biological behavior contributing to both. In this study, we found that for those with small BC, younger age, higher tumor grades, larger tumor sizes, higher Ki67 levels, TNBC, and HER2+ subtypes were associated with the administration of adjuvant chemotherapy, which is consistent with actual treatment recommendations. However, among the whole population, patients had no clear benefit from chemotherapy, chemotherapy did not increase the iDFS or BCSS, and tumor size could not predict the benefit of chemotherapy. Furthermore, we found that the analysis based on molecular subtype was statistically significant, which supports that molecular subtype was a determinant predictive factor of chemotherapy benefit for those small BCs.

Regarding HR+/HER2− BCs, it is known that several genomic signatures, such as Oncotype DX and MammaPrint, have become important tools in determining the risk of recurrence in HR+/HER2− patients as well as the benefit of chemotherapy. For example, the National Comprehensive Cancer Network (NCCN) guidelines recommend a performance of a 21-gene recurrence score to predict the benefit of chemotherapy for HR+/HER2− T1N0 BC patients, especially for tumors more than 0.5 cm in size (23). Patients with an intermediate or high score should consider adjuvant chemotherapy; if the 21-gene recurrence score is absent, clinicians should take chemotherapy into consideration. However, our result revealed that little benefit of chemotherapy was observed among those small, node-negative HR+/HER2− BCs, which could be considered when deciding whether to omit chemotherapy. Combined with the good prognosis of small HR+/HER2− BCs, endocrine treatment might be sufficient for most of this population. On the other hand, our result suggests that the value of those genomic signature tools in those HR+/HER2− small BC patients is still uncertain, and prognostic genomic signature tests are likely unnecessary for these patients. To further investigate the value of genomic signature and the question of who needs adjuvant chemotherapy among those HR+/HER2− small BC patients, clinical trials are needed.

TNBC, which is defined as negative hormonal receptor and HER2 status, accounts for nearly 20% of all BCs and has an aggressive biological behavior (24). Our study confirmed that even these small, node-negative TNBC tumors had an increased recurrence risk and BC-related death compared with other subtypes. Because TNBC tumors do not respond to endocrine treatment and anti-HER2 therapy, chemotherapy remains the only option available (25, 26). Current guidelines generally recommend adjuvant chemotherapy for TNBC patients with tumor size >0.5 cm (23). In our study, trends suggested a distinct benefit of iDFS survival with chemotherapy in T1N0 TNBC patients. Taking the high risk of recurrence and the need for improvements of prognosis into consideration, our results suggest that adjuvant chemotherapy should be considered even for those small TNBC tumors, and more clinical trials are warranted to investigate new treatment patterns (e.g., immune therapy) for these small TNBCs.

For HER2+ tumors, numbers of randomized clinical trials have shown that trastuzumab added to chemotherapy could improve survival in the adjuvant setting (27–29). However, few patients with T1N0 HER2+ tumors, especially tumor size <1 cm, were recruited in these trials. Despite this fact, since 2010, the NCCN guidelines have recommended that chemotherapy and trastuzumab should be considered for offering to HER2+ T1bN0 patients (23). Thus, most clinicians recommend HER2-targeted therapies for these small tumors because HER2+ BC has an increased recurrence risk and due to the generally low toxicity of anti-HER2 agents, such as trastuzumab. In this study, which included 427 HER2+ small tumors, we found a trend of benefit from adjuvant chemotherapy with or without trastuzumab. Combined with the APT trial’s results, our results support that single-agent chemotherapy plus trastuzumab could be considered an attractive approach for small, node-negative HER2+ BC, balancing benefits *versus* risks (30).

However, our study has several limitations. First, since the present study was retrospective, the baseline characteristics and treatment were not randomized, which makes it difficult to conclude whether survival data reflect the response to adjuvant chemotherapy or the natural history of specific subgroups. Second, the median follow-up period for our cohort was 47.9 months, which was relatively short for small BCs, especially for HR+/HER2− patients. Due to relatively little events, a longer follow-up time will guarantee the reliability of our findings. Moreover, the classification of chemotherapy *versus* non-chemotherapy did not account for the impact of the variability of chemotherapy regimens.

## Conclusion

In summary, our study shows that among patients with pT1N0 BC, a group with generally favorable clinical outcomes, molecular subtype was a significant prognostic factor, and TNBC had the worst prognosis. Furthermore, T1N0 BC patients could not clearly benefit from adjuvant chemotherapy, which was potentially beneficial for only TNBC and HER2+ patients. Therefore, compared with tumor size, the molecular subtype of BC may facilitate a more accurate tailoring of treatment recommendations for T1N0 BC patients and guide possible clinical de-escalating therapy.

## Data Availability Statement

The raw data supporting the conclusions of this article will be made available by the authors, without undue reservation.

## Ethics Statement

Written informed consent was obtained from the individual(s) for the publication of any potentially identifiable images or data included in this article.

## Author Contributions

SZ: writing—original draft, visualization, investigation, and funding acquisition. YL: investigation. WC: investigation and resources. XF: investigation. XC: writing—reviewing and editing, project administration, and supervision. KS: supervision, project administration, and funding acquisition. All authors contributed to the article and approved the submitted version.

## Funding

This study was financially supported by grants from National Natural Science Foundation of China (grant number: 81472462 and 81772797), Shanghai Municipal Education Commission Gaofeng Clinical Medicine Grant Support (20172007), Scientific Research Project of Shanghai Municipal Health Commission (20194Y0419), and Ruijin Youth NSFC Cultivation Fund (2019QNPY02013).

## Conflict of Interest

The authors declare that the research was conducted in the absence of any commercial or financial relationships that could be construed as a potential conflict of interest.

## Supplementary Material

Supplementary Figure 1 Annual risk curve of iDFS and BCSS according to molecular subtype. a) Annual risk curve of iDFS according to molecular subtype. b) Annual risk curve of BCSS according to molecular subtype.
