# Supplementary material for: Molecular Subtype May Be More Associated With Prognosis and Chemotherapy Benefit Than Tumor Size in T1N0 Breast Cancer Patients: An Analysis of 2,168 Patients for Possible De-Escalation Treatment
Source: Front Oncol. 2021 Feb 19;11:636266. doi: 10.3389/fonc.2021.636266 (PMC7933524; doi:10.3389/fonc.2021.636266)
Supplement: Supplementary file 2 [file Table_1.docx]

Supplementary Table 1

Univariate analyses of chemotherapy administration according to tumor characteristics

| Characteristics | Chemotherapy  YES(N=1080) NO(N=1088) | | Univariate  χ^2^ P |
| --- | --- | --- | --- |
| Age (median age) | 52 | 60 | 199.19 <0.001 |
| <50  50-65  65+ | 422(60.2%)  554(56.2%)  104(21.6%) | 279(39.8%)  432(43.8%)  377(78.4%) |  |
| Menopasual status | |  | 43.07 <0.001 |
| Premenopausal  Postmenopausal | 465(59.2%)  615(44.5%) | 321(40.8%)  767(55.5%) |  |
| Histological grade | |  | 377.17 <0.001 |
| I  II  III  NA | 32(18.8%)  480(47.4%)  459(81.2%)  109(25.9%) | 138(81.2%)  532(52.6%)  106(18.8%)  312(74.1%) |  |
| Pathological type | |  | 77.05 <0.001 |
| IDC  ILC  Others | 994(53.7%)  25(33.3%)  61(25.3%) | 858(46.3%)  50(66.7%)  180(74.7%) |  |
| Tumor size |  |  | 146.97 <0.001 |
| T1a  T1b  T1c | 85(24.7%) 188(41.1%)  807(59.0%) | 259(75.3%)  269(58.9%)  560(41.0%) |  |
| ER status |  |  | 200.74 <0.001 |
| Positive  Negative | 666(41.0%)  414(76.1%) | 958(59.0%)  130(23.9%) |  |
| PgR status |  |  | 258.49 <0.001 |
| Positive  Negative | 490(36.4%)  590(72.0%) | 858(63.6%)  230(28.0%) |  |
| HER2 status |  |  | 194.99 <0.001 |
| Positive  Negative | 342(80.1%)  738(42.4%) | 85(19.9%)  1003(57.6%) |  |
| Ki67 level | |  | 402.68 <0.001 |
| <14%  ≥14% | 273(26.9%)  807(70.1%) | 743(73.1%)  345(29.9%) |  |
| Molecular subtype | |  | 357.70 <0.001 |
| HR+/HER2-  HER2+  TN | 507(35.3%)  342(80.1%)  231(75.5%) | 928(64.7%)  85(19.9%)  75(24.5%) |  |

**Abbreviations:**

*Uv*, univariate; *IDC,* Invasive ductal carcinoma; *ILC,* Invasive lobular carcinoma; *ER,* Estrogen receptor; *PgR,* Progesterone receptor; *HR,* *Hormonal* receptor; *HER2,* Human epidermal growth factor receptor-2; *TN,* Triple negative; *NA*, not available

Supplementary Table 2

| Regimens |  | Total | HR+/HER2-  T1a T1b T1c | | | T1a | HER2+  T1b | T1c | T1a | TN  T1b | T1c |
| --- | --- | --- | --- | --- | --- | --- | --- | --- | --- | --- | --- |
| T1a |  |  |  |  |  |  |  |  |  |  |  |
| CMF  Anthracycline without Taxane  Taxane and Cyclophosphamide  Anthracycline Plus Taxane  Single chemotherapy agent  Others | | 10  148  423  409  58  32 | /  1  5  /  1  3 | 1  28  60  6  1  4 | 2  84  245  58  2  6 | /  5  15  8  27  8 | /  3  15  28  7  1 | /  11  48  148  13  5 | /  3  5  2  1  3 | 3  4  10  17  /  / | 4  9  20  142  6  2 |

Regimens of chemotherapy according to tumor subtype and tumor size

**Abbreviations:**

*HR,* *Hormonal* receptor; *HER2,* Human epidermal growth factor receptor-2; *TN,* Triple negative
